# Vascular supply of the metacarpophalangeal joint

**DOI:** 10.3389/fmed.2022.1015895

**Published:** 2022-10-20

**Authors:** Gabor Baksa, Kalman Czeibert, Veronika Sharp, Stephan Handschuh, Janos Gyebnar, Laszlo Barany, Szabolcs Benis, Gabor Nyiri, Peter Mandl, Ors Petnehazy, Peter Vince Balint

**Affiliations:** ^1^Laboratory for Applied and Clinical Anatomy, Department of Anatomy, Histology and Embryology, Semmelweis University, Budapest, Hungary; ^2^Department of Ethology, Institute of Biology, Eötvös Loránd University, Budapest, Hungary; ^3^Division of Rheumatology, Department of Medicine, Santa Clara Valley Medical Center, San Jose, CA, United States; ^4^VetCore Facility for Research, University of Veterinary Medicine Vienna, Vienna, Austria; ^5^Medical Imaging Centre, Faculty of Medicine, Semmelweis University, Budapest, Hungary; ^6^Department of Neurosurgery, University of Erlangen-Nürnberg, Erlangen, Germany; ^7^Department of Orthopedic Surgery and Traumatology, Ghent University Hospital, Ghent, Belgium; ^8^Laboratory of Cerebral Cortex Research, Institute of Experimental Medicine, Budapest, Hungary; ^9^Division of Rheumatology, Department of Internal Medicine III, Medical University Vienna, Vienna, Austria; ^10^Medicopus Non-profit Ltd, Kaposvar, Hungary; ^11^Justanatomy Ltd, Kaposvar, Hungary; ^12^Károly Rácz Doctoral School of Clinical Medicine, Semmelweis University, Budapest, Hungary; ^13^3rd Department of Rheumatology, National Institute of Rheumatology and Physiotherapy, Budapest, Hungary

**Keywords:** arterial supply, articular, Doppler, metacarpophalangeal joint, rheumatoid arthritis, ultrasound

## Abstract

**Objective:**

To describe in detail the arterial vasculature of metacarpophalangeal joints 2–5 on cadaver specimens and to compare it to ultrasound imaging of healthy subjects.

**Methods:**

Eighteen hands of donated human cadavers were arterially injected and investigated with either corrosion casting or cryosectioning. Each layer of cryosectioned specimens was photographed in high-resolution. Images were then segmented for arterial vessels of the metacarpophalangeal (MCP) joints 2–5. The arterial pattern of the joints was reconstructed from the segmented images and from the corrosion cast specimens. Both hands of ten adult healthy volunteers were scanned focusing on the vasculature of the same joints with high-end ultrasound imaging, including color Doppler. Measurements were made on both cryosectioned arteries and Doppler images.

**Results:**

The arterial supply of MCP joints 2–5 divides into a metacarpal and a phalangeal territory, respectively. The metacarpal half receives arteries from the palmar metacarpal arteries or proper palmar digital arteries, while the phalangeal half is supplied by both proper and common palmar digital arteries. Comparing anatomical and ultrasonographic results, we determined the exact anatomic location of normal vessels using Doppler images acquired of healthy joints. All, except three branches, were found with less than 50% frequency using ultrasound. Doppler signals were identified significantly more frequently in MCP joints 2–3 than on 4–5 (*p* < 0.0001). Similarly, Doppler signals differed in the number of detectable small, intraarticular vessels (*p* < 0.009), but not that of the large extraarticular ones (*p* < 0.1373). When comparing measurements acquired by ultrasound and on cadaver vessels, measurements using the former technique were found to be larger in all joints (*p* < 0.0001).

**Conclusion:**

Using morphological and ultrasonographic techniques, our study provides a high-resolution anatomical maps and an essential reference data set on the entire arterial vasculature of healthy human MCP 2–5 joints. We found that Doppler signal could be detected in less than 50% of the vessels of healthy volunteers except three locations. Intraarticular branches were detected with ultrasound imaging significantly more frequently on healthy MCP 2–3 joints, which should be taken into account when inflammatory and normal Doppler signals are evaluated. Our study also provides reference data for future, higher-resolution imaging techniques.

## Introduction

Inflammation of tissues and/or vessels of different location and size is a major component of common rheumatological pathologies such as synovitis, enthesitis and vasculitis (1–4). Metacarpophalangeal (MCP) joints are frequently involved in inflammatory arthritides especially in rheumatoid arthritis (RA), psoriatic arthritis (PsA), and juvenile idiopathic arthritis (5–7). Additionally, finger arteries are often affected in various types of vasculitides (3). Color-, power Doppler, and B (brightness)-flow ultrasonography can visualize the blood flow inside vessels of different sizes (8). Spectral Doppler can depict this same blood flow in a graph (9). Advanced imaging applications targeting microvascular imaging are evolving techniques (10, 11), which can detect velocity of blood cells in real-time, while contrast-enhanced ultrasound is based on detecting intravenous microbubbles (12). Ultrasound is capable of recording both still images or videos of musculoskeletal tissue in various regions during static or dynamic examination (13). However, ultrasound has its own limitations. Without an acoustic window or without adequate sensitivity for small vessels or for slow flow, ultrasound is not capable of detecting flow signal (8). All limitations and advantages considered, ultrasound has a better resolution but is much less sensitive for detecting color than the human eye. Most humans are trichromats and are able to distinguish 10 million shades of color while a typical high-end ultrasound unit can display only around 256 shades (14–16).

Ultrasonography is commonly used by rheumatologists to detect pathological blood flow in a plethora of subclinical or clinical rheumatic and musculoskeletal disease (RMD) or to document diminished flow in cases of Raynaud phenomenon (5, 17, 18). However, one needs to be cognizant that nowadays high-end ultrasound equipment is also capable of detecting normal blood flow (with some limitations) in healthy or asymptomatic joints (19, 20).

Operator dependency has been an obstacle to rheumatological ultrasonography. For proficiency in ultrasonography not only proper image acquisition but correct interpretation is paramount. Besides a deep understanding of ultrasound physics and equipment operation techniques, a high level of anatomical and pathological knowledge is crucial to perform musculoskeletal ultrasonography appropriately. While large vessel anatomy of the hand is routinely taught at medical courses, small vessel anatomy is usually only included in teaching material for hand surgical specialties (21).

The aim of this study was to map the arterial vasculature of MCP joints 2–5 using hand corrosion casts and cryosectioning from injected cadaver specimens, two validated anatomical techniques (22). In addition, we used musculoskeletal ultrasound investigation of healthy individuals, a readily accessible, patient-friendly imaging technique, in order to provide an atlas, which would facilitate distinguishing Doppler flow in healthy vessels from pathological signal seen in RMDs.

## Materials and methods

### Source and preparation of cadaveric specimens

Cadaveric specimens with post mortem time 1–4 days were harvested from donated bodies at the Department of Anatomy, Histology and Embryology, Semmelweis University, Budapest, Hungary. Body donation is permitted and controlled by Section 222 of Chapter 12 of Act CLIV on Health 1997 and by Senate’s decree Act 110/2020. (VII.07.) “Handling procedures of donated human material (body/organ/tissue).” Hands of female and male cadavers were separated 7–10 cm above the wrist. Both the radial and the ulnar arteries were identified, cannulated and irrigated with saline. At this step both hands of one male and one female cadaver were further prepared for cryosectioning, while the remaining hands underwent corrosion casting.

#### Cryosectioning

The arteries were injected with Vytaflex 20^®^ (Smooth-On Inc., Macungie, PA, USA) polyurethane colored with So Strong^®^ (Smooth-On Inc., Macungie, PA, USA) red tint. Following a 24-h hardening time at 4°C the hands were placed on −30°C. After the hands were frozen, four blocks each containing one region-of-interest (MCP 2–5) were cut out using a band saw. The blocks extended from the middle of the proximal phalanx of the 3rd finger distally to the middle of the metacarpal bone of the thumb proximally in the axial plane. All four metacarpal blocks per cadaver were positioned in one plastic container facing palmar side down, then embedded in porcine gelatin (G2500-500G, gel strength 300, Type A) (SIGMA-ALDRICH Chemie GmbH, Steinheim, Germany) and were kept on −80°C after the gelatin hardened. Cryosectioning was carried out with the plastic container attached to a CNC milling machine (NCT Kondia 640B, NCT, Budapest, Hungary; rotational speed 3,000 rpm, cutter diameter 200 mm, feed rate 800 mm per revolution). The layer thickness of milling was 50 μm. At every milling step the fresh surface was photographed with a Canon EOS 5DS camera at 8,688 × 5,792 pixels resolution per image. Images were then processed using Adobe Photoshop CS3^1^ and Thermo Fisher Scientific Amira for Life Sciences 6.1 software.^2^ Since the subsequent image segmentation step in Amira required a 8-bit grayscale volume, we used a Photoshop algorithm to convert the RGB (Red Green Blue) volume to a grayscale image stack, while maintaining the high contrast of the arteries using a selective red color channel subtraction. The grayscale images were then imported into Amira. Possible minor image dislocations were corrected with the “Align Slices” module. Subsequently, using the “Segment Editor” panel of the “Edit New Label Field” module, semi-automatic segmentation was performed to model the arteries. Measurements were taken at predefined locations detailed by the results (section “Measurements on cryosectioned specimens”). The applied technique of cryosectioning, including the steps of image processing and segmentation of the vessels is described in an earlier publication by our group (22).

#### Corrosion casting

The remaining hands were injected with ACRIFIX 190 (2 R 0190) (Evonik Industries AG., Germany) colored with red Akemi Akepox coloring paste (AKEMI GmbH., Nurnberg, Germany) and catalyzed with Betox 50-PC hardener (Oxytop Sp. z o.o., Stęszew, Poland). Following a 24-h hardening time, the hands were put separately in 2 L plastic containers filled with tap water and adjusted with two Somat Gold 12 Actions (Henkel AG., Germany) dishwasher tablets. The specimens were kept in this solution at +36°C for 6–10 weeks. The solution was changed every 2–3 weeks, while the specimens were handled with great care to avoid fracture of the intermediate corrosion casts due to potential tearing caused by movement of the soft tissue mass. After all soft tissue was digested, the specimens were carefully washed and left in cold water for 3 days to eliminate the remaining chemicals and odor. The vascular pattern of each MCP joint 2–5 were investigated visually and if needed with a Wild Heerbrugg M5A stereomicroscope (Wild Heerbrugg Switzerland Microscope, Switzerland) using 12–50× magnification. All findings were documented using a Canon EOS 5D digital camera, Macro Ring Lite MR-14EX flash and 50, 100, and 65 mm macro lenses (CANON Inc., Tokyo, Japan), respectively.

### Ultrasound examination

#### Study participants

All examined persons were asymptomatic without current diagnosis of rheumatic and musculoskeletal hand disease. MCP joints 2–5 of both hands were scanned for vascular signals using an ultrasonography machine (GE Logiq E9, General Electric Company, Boston, MA, USA) equipped with a small-footprint high-frequency ultrasound transducer (GE L8-18i).

#### Scanning method

Before scanning a joint, patients were asked to place their examined hand in a tap water bath measuring 38°C as confirmed by a thermometer for 4 min to eliminate confounding of outdoor temperature and of individual temperature variance of the hands. No other vasodilating method or agent was used. For scanning, hands were first positioned palm down, fingers extended and slightly abducted, after which hands were placed palm up, with the fingers kept in the same position. Scanning was carried out on both the palmar and dorsal side of each examined joint, and additionally on the radial side of the 2nd and on the ulnar side of the 5th MCP joints, respectively. The ultrasound machine was used in color Doppler mode. The parameters for color Doppler were 11.9 MHz, PRF 0.6, WF 54, and the Doppler box was set to maximal size in both the horizontal and vertical planes and gain was reduced until artifacts disappeared. The settings were kept unchanged for each ultrasound examination except for the value of the color gain which was adjusted if needed within a very narrow range (15.5–19). The ultrasound transducer was held parallel to the force bearing axis of the metacarpal bone on every side of the examined joint. Each joint was scanned from the radial to the ulnar margin. Additionally, the radial side of the 2nd and the ulnar side of the 5th metacarpal joints were scanned from the dorsal to the palmar surface. Special care was taken to use abundant gel and to avoid compression to prevent temporary closure of smaller blood vessels.

#### Image interpretation and measurement

Doppler signals were interpreted as valid if both of the following criteria were met: (1) the localization of the Doppler signal had to match a vessel on both cryosectioned and corrosion cast anatomical specimens; (2) reverberating Doppler signals were excluded. The strength and extension of the Doppler signal had no influence on the decision of validity. Measurements were taken on the shortest diameter of the vascular signals.

### Statistical analysis

The statistical analysis was performed using the R Software (version 4.0.3). The level of the statistical significance was set at *p* = 0.05. Fisher’s exact test was used for comparing categorical variables. Continuous variables were compared using two-sampled *t*-test after confirming their normal distribution using Shapiro–Wilk test.

## Results

### Cadaveric specimens

Eight right and six left hands of 8 female cadavers (ages 55–84 years, mean 69.5 ± 14.5 years) and three right and two left hands of 4 male cadavers (ages 48–94 years, mean 71 ± 23 years) were used for anatomical preparation. The arteries of both hands of a 48-year-old male and a 55-year-old female cadaver were used for cryosectioning while the remaining hands were used for corrosion casting. The number of joints used for each technique are shown in Table 1. Seven joints (1 MCP1, 1 MCP2, 2 MCP4, and 3 MCP5 joint) were excluded from the anatomical techniques either due to damage during the preparation procedure (corrosion casting, n:3) or due to failed injection (cryosectioning, n:4). Based on the corrosion casting and cryosectioning results we divided the arterial supply into metacarpal (proximal half) and phalangeal (distal half) territories, respectively. As many corrosion cast specimens suffered partial injuries of the phalangeal territory despite careful handling, the blood supply of the distal half of the metacarpal joints was investigated only on cryosectioned specimens. However, the total number of investigated joint specimens was large enough in both territories to determine their general arterial pattern, and to compare it with ultrasound imaging results.

**TABLE 1:** Number of metacarpophalangeal joints 2–5 by method of investigation.

Investigation method	MCP2	MCP3	MCP4	MCP5
Corrosion casting^a^	15	15	14	13
Cryosectioning	3	3	3	3
Ultrasound	20	20	19	18
Total number of joints	38	38	36	34

MCP, metacarpophalangeal joint.

^a^Investigated only for the blood supply of the metacarpal territory.

#### Arterial supply of the metacarpal territory on cadaveric specimens

**FIGURE 1:**
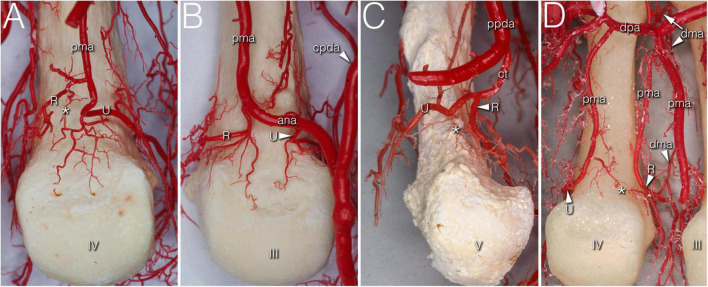
Variations of R- and U- branches on corrosion cast specimens (palmar view). **(A)** Dominant U-branch as a continuation of the palmar metacarpal artery. Note the two palmar enosseal vessels originating as a common trunk from the U-branch. **(B)** The U-branch originates from the anastomosis between the palmar metacarpal artery and the common palmar digital artery. **(C)** The R- and U-branches originate as a common trunk from the 10th proper palmar digital artery. **(D)** The R-branch originates from the early dividing neighboring palmar metacarpal artery. The U-branch is the continuation of a separately originating palmar metacarpal artery. Note the dorsal metacarpal artery anastomosing with the R-branch. ana, anastomosis between the palmar metacarpal artery and the common palmar digital artery; *, anastomosis between R and U branch; cpda, common palmar digital artery; ct, common trunk; dma, dorsal metacarpal artery; dpa, deep palmar arch; pma, palmar metacarpal artery; ppda, proper palmar digital artery; R, R-branch; U, U-branch; III, IV, V: 3rd, 4th, and 5th metacarpal heads.

In general, for each metacarpal joint we found two main supplying arteries arising from the palmar side, each giving off further articular branches. One vessel ran toward the radial and the other vessel toward the ulnar side. Therefore, we labeled these “R-branch” and “U-branch,” respectively. Both the R- and U-branch were present as single vessels in 69/69 (100%) joints investigated with corrosion casting or cryosectioning. Usually they originated from the palmar metacarpal arteries (PMA) of the deep palmar arch (DPA), except for the MCP5 joint, where the proper palmar digital artery (PPDA) was the most frequent source. Representative images of the anatomical variants are shown on Figure 1, their distribution in Table 2.

**TABLE 2:** Varying origins of the radial and ulnar branches based on localization.

		MCP2 *n* = 18 (%)	MCP3 *n* = 18 (%)	MCP4 *n* = 17 (%)	MCP5 *n* = 16 (%)
R-branch	PMA (%)	**13** (72.2)	**5 + 13**^c^ (100)	**13 + 1**^c^ (82.4)	**5** (31.25)
	PPDA (%)	**5**^a^ (27.8)	0	0	**10** (62.5)
	DMA (%)	0	0	**3**^d^ (17.6)	**1**^d^ (6.25)
U-branch	PMA (%)	**11** (61.1)	**5 + 13**^c^ (100)	**16 + 1**^c^ (100)	**4** (25.0)
	PPDA (%)	0	0	0	**12** (75.0)
	PMA/CPDA anastomosis (%)	**7**^b^ (38.9)	0	0	0

CPDA, common palmar digital artery; DMA, dorsal metacarpal artery; PMA, palmar metacarpal artery; PPDA, proper palmar digital artery; R, radial; U, ulnar.

^a^If no princeps pollicis artery is present, then this shows the origin from the 3rd proper palmar digital artery.

^b^Common palmar digital artery supplied partially or completely by the palmar metacarpal artery.

^c^Early division or separate (‘doubled’) origin of palmar metacarpal artery.

^d^Main supply from the dorsal metacarpal artery or its collateral branch, both of them derived from the deep palmar arch. Proximally thin origin from the palmar metacarpal artery.

**FIGURE 2:**
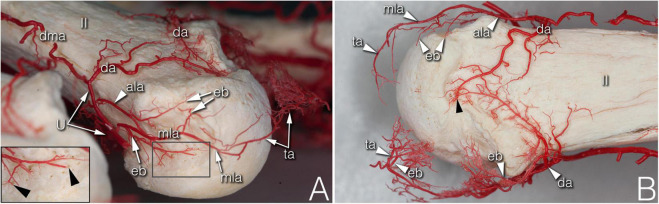
Supplying branches to the metacarpal territory on corrosion cast specimens. **(A)** (right hand anterolateral view) A strong enthesial branch arises directly from the first part of the main lateral artery and gives off branches running toward the nutrient foramina of the lateral depression (see insert). Both the upper accessory lateral artery and the main lateral artery give off one further enthesial branch to the upper part of the lateral depression. The main lateral artery curves to and terminates at the projection of the triangular arcade. **(B)** (upper view of the same specimen) Note the spiky character of the ulnar sided enthesial branches (top of image), while radially (bottom of image) both the enthesial branch and the triangular arcade artery demonstrate a bushy appearance. ala, accessory lateral artery; da, dorsal arcade; dma, dorsal metacarpal artery; eb, enthesial branch; mla, main lateral artery; ta, triangular arcade; U, U-branch; black arrowheads, entry point of enosseal arteries; II, 2nd metacarpal head.

**FIGURE 3:**
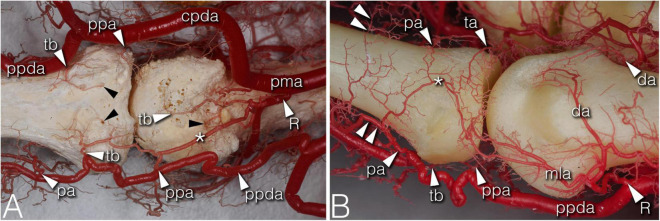
Arteries of the phalangeal territory on corrosion cast specimens. **(A)** (4th finger, palmar view) Note the long anastomosis between the R-branch and the palmar plate artery. Proximal from the anastomosis a short trunk arises acting as a tenosynovial vessel, with one branch reaching to the metacarpal head and another branch extending to the projection of the flexor tendon. **(B)** (2nd finger, dorsolateral view) The phalangeal arcade originates as one common trunk, with one branch supplying the arcade on the dorsal surface of the phalanx, and another branch forming a more superficial arcade according to the projection of the extensor hood. The triangular and phalangeal arcade are in connection through a short anastomosis. Note the small branches from the triangular arcade showing a pectinate character in the axial plane. *, anastomosis; cpda, common palmar digital artery; da, dorsal arcade; mla, main lateral artery; pa, phalangeal arcade; pma, palmar metacarpal artery; ppa, palmar plate artery; ppda, proper palmar digital artery; R, R-branch; ta, triangular arcade; tb, tenosynovial branch; black arrowheads, entry point of enosseal arteries; doubled arrowheads, supplying arch for the extensor tendon.

**FIGURE 4:**
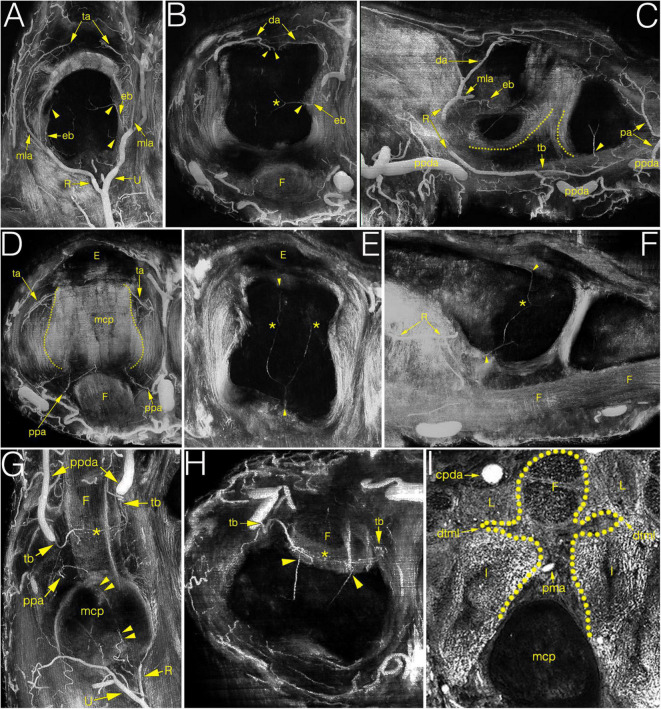
Multiplanar (MIP) image reconstructions on cryosectioned specimens demonstrating the relationship between arteries and joint structures. **(A–C)** main supplying branches on coronal, axial and sagittal plane, respectively. Note on C the elongated anastomosis between the R-branch and the tenosynovial branch. Dotted lines indicate the palmar articular surfaces of bones; **(D)** palmar plate artery and the triangular arcade on the axial plane. Dotted lines indicate the lateral borders of metacarpal head; **(E,F)** blood supply of the metacarpal head on the axial and sagittal plane. Note the anastomosis between the dorsal, palmar and lateral enosseal arteries, respectively. Compare with panel **(B)**. **(G,H)** origin and course of the tenosynovial trunk on the coronal and axial plane. Note the anastomosis between the two sides within the flexor tendon. **(I)** homuncule shaped (dotted line) soft tissue complex in the center with the palmar metacarpal artery on the axial plane. *, anastomosis; cpda, common palmar digital artery; da, dorsal arcade; dtml, deep transverse metacarpal ligament; eb, enthesial branch; E, extensor tendon; F, flexor tendon; I, interosseous muscle; L, lumbrical muscle; mcp, metacarpal; mla, main lateral artery; pa, phalangeal arcade; pma, palmar metacarpal artery; ppa, palmar plate artery; ppda, proper palmar digital artery; R, R-branch; ta, triangular arcade; tb, tenosynovial trunk; U, U-branch; arrowheads, entry point of enosseal arteries; doubled arrowheads, supplying artery from the R-branch to the flexor tendons.

In numerous cases, the R- and U-branches formed connections to the DMA or its collateral branch parallel to the metacarpal shaft. Specific examples are connections at the MCP5 joint to the carpal rete and simultaneous anastomoses with the vessels listed above. In total, single or multiple anastomoses were seen depending on joint location radially in 60–88.89% of cases, ulnar in 61.11–82.35% of cases (these ranges represent the different probabilities of localizations on MCP2-MCP5, Figures 1D, 2A). Coursing further on the lateral surface of metacarpal heads, both the R- and U-branches gave off a single and strong forward running artery in 88.41% of the joints. In the remaining 11.59% of the joints the same was found, only either radially or ulnar, except one case of bilateral absence. Parallel to this vessel, which we labeled the “main lateral artery” (MLA), a shorter “accessory lateral artery” (ALA) was detected radially in 12 joints (17.39%) and ulnar in 12 joints (17.39%). These originated either from the MLA, or directly from the R- and U-branches below or above the origin of the MLA. A last segment of the R- and U-branches curving on to the dorsal surface of the metacarpal head was recognized radially in 81.25–94.44%, on the ulnar side in 72.22–94.44% of joints, respectively. In 49.28% they formed an anastomosis immediately according to the dorsal depression, which we labeled the “dorsal arcade” (DA) (Figures 2, 3B, 4A–C). Simultaneously, in five specimens (7.25%) an anastomosis was detected between the R- and U-branches. In these cases, a complete arterial ring was present around the metacarpal head. The frequency of these findings is summarized in Table 3.

**TABLE 3:** Occurrence of the main lateral arteries, accessory lateral arteries and dorsal arcade at metacarpophalangeal joints 2–5.

Σ_n_ = 69		MCP2 *n* = 18 (%)	MCP3 *n* = 18 (%)	MCP4 *n* = 17 (%)	MCP5 *n* = 16 (%)
MLA	Bilateral(%)	**17** (94.4)	**16** (88.9)	**15** (88.2)	**13** (81.25)
	Radial (%)	–	**2** (11.1)	–	–
	Ulnar (%)	**1** (5.6)	–	**2** (11.8)^a^	**2** (12.5)
	Absent (%)	–	–	–	**1** (6.25)
ALA	Radial (%)	**3** (16.7)	**2** (11.1)	**3** (17.7)	**4** (25.00)
	Ulnar (%)	**1** (5.6)	**1** (5.6)	**3** (17.7)	**7** (43.8)
DA	Present (%)	**10** (55.6)	**8** (44.4)	**8** (47.1)	**8** (50.0)
	Absent (%)	**8** (44.4)	**10** (55.6)	**9** (52.9)	**8** (50.0)
	Multiple (%)	**4** (22.2)	–	**2** (12.5)	**4** (28.6)

^a^In one case the radial main lateral artery was absent, but the territory of it was from a deep seated dorsal metacarpal artery supplied.

ALA, accessory lateral artery; DA, dorsal arcade; MCP, metacarpophalangeal joint; MLA, main lateral artery.

The bold values describe the number of joints based on vascular findings and localization.

During their course, the R- and the U-branches, the MLA and the ALA, respectively, give off radially (64.29–94.12%), or on the ulnar side (70.59–100.00%) 1–4 small branches to the hollow lateral surface of the metacarpal head, which we labeled the “lateral depression” (Figure 2). We investigated the MLA and ALA in the coronal plane of the cryosectioned specimens. In all cases these ran on the outer surface of a triangular shaped enthesis over the metacarpal head and the base of the proximal phalanx. The small branches to the lateral depression penetrated this enthesis. Therefore, we labeled them “enthesial branches” (Figures 4A–C).

The MLA then curved radially (50.00–62.50%) or ulnar (38.89–76.47%) into the space in the dorsal compartment between the articulating bones and terminated there with or without anastomosing with the contralateral MLA (Figures 2A,B). These terminal segments of the MLA were consequently found to supply the dorsal triangle on the cryosectioned specimens (Figures 4A,D). Independent from the presence or absence of an anastomosis, we labeled these “triangular arcades.” In two isolated cases several small perpendicular branches were detected along this arcade showing a pectinate character in the axial plane (Figure 3B).

The most terminal arteries supplied the metacarpal heads. These appeared most frequently at the dorsal depression (1–7 vessels, 58.82–73.33%) coming from the dorsal arcade or, in case no anastomosis was present, from the terminal part of the R- and U-branches, respectively (Figure 2B). The second most frequent occurrence was found on the ulnar aspect (1–7 vessels, 26.67–66.67%) (Figure 2A). The third most common occurrence was on the palmar surface (1–5 vessels, 26.67–55.56%) (Figures 1A, 3A). The radial side showed the lowest occurrence (1–3 vessels, 17.65–27.78%). Both the radial and ulnar arteries originated from enthesial branches (Figures 4A–C), while the palmar ones originated directly from the R- and U-branches (Figures 1A, 4F). In cryosectioned specimens enosseal anastomoses of these arteries were also detected (Figures 4B,E,F).

#### Arterial supply of the phalangeal territory on cadaveric specimens

The main supplying vessels were the PPDAs and the CPDAs of each finger, respectively. In general, a short trunk was observed radially (66.67%) or ulnar (58.33% of all cases) either separate from the PPDAs or originating from the bifurcation point of CPDAs supplying the palmar plate. Therefore, we labeled these the “palmar plate arteries” (PPA) (Figure 4D).

Distally, the PPDAs gave off a second artery radially (83.33%) or ulnar (100%), which then branched into a long and thin, forward running vessel penetrating the flexor tendon sheaths, while its other branch coursed medially and backward to supply the palmar surface of the proximal phalanx’s base. We labeled this artery “tenosynovial branch” (Figures 4G,H). In one case, the radial tenosynovial branch of the index finger originated directly from the R-branch and served small branches also to the palmar plate (Figure 4C). A third relevant branch was detected radially (50.00%) directly from the PPDA, but ulnar (41.67%) from the tenosynovial branch. These ran to the dorsal side of the base of the proximal phalanx, where—independently from their origin - these branches anastomosed with the contralateral ones creating an arterial arch in 75.00% of the cases. The latter was labeled “phalangeal arcade” (Figures 3, 4C). In the remaining cases, where present, we observed the phalangeal arcade originating bilaterally from the MLA (16.67%).

**FIGURE 5:**
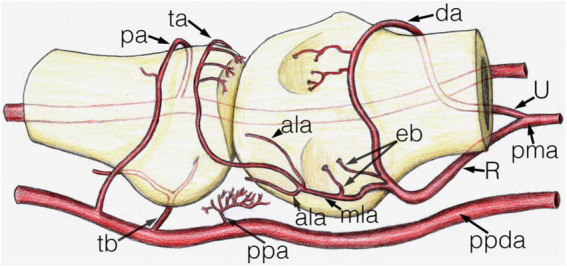
Schematic drawing of the main arterial vessels of the metacarpophalangeal joint. The proximal (metacarpal) half and distal (phalangeal) half are shown on the right and left side of the image respectively. ala, accessory lateral artery; da, dorsal arcade; eb, enthesial branch; mla, main lateral artery; pa, phalangeal arcade; pma, palmar metacarpal artery; ppa, palmar plate artery; ppda, proper palmar digital artery; R, R-branch; ta, triangular arcade; tb, tenosynovial branch; U, U-branch.

Arteries with detectable size supplying the base of the proximal phalanx were detected only on the palmar and dorsal surfaces of the bone. On the palmar surface, symmetrically (both on the radial and ulnar side) one artery penetrated the bony cortex (83.33%). In one case, two arteries were observed radially. In one additional joint no supplying branch was found. The palmar vessels originated from the tenosynovial branch (Figures 3A, 4C,H). Dorsal phalangeal arteries were identified only in 50.00% of the joints branching directly from the phalangeal arcade. In 41.67% we found these only radially, in one case bilaterally. A schematic drawing about the general arterial pattern of metacarpophalangeal joints 2–5 is shown in Figure 5.

#### Measurements on cryosectioned specimens

**FIGURE 6:**
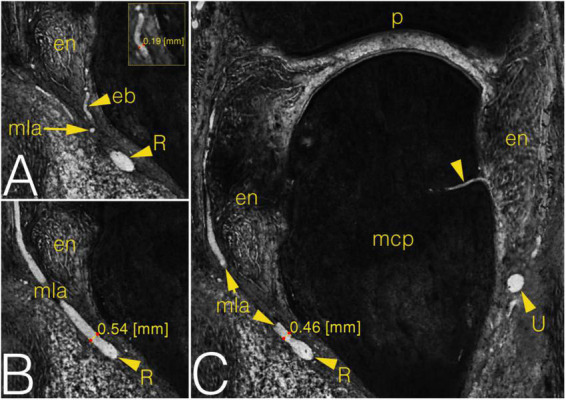
Grayscale images of a cryosectioned right MCP2 joint demonstrating joint anatomy and measurement on the arteries in coronal plane. **(A)** Corresponds to the left inferior quadrant of panel **(C)**, level of cryosectioning 13 layers (0.65 mm) palmar. Insert shows diameter measurement on enthesial branch. **(B)** Corresponds to the left inferior quadrant of panel **(C)**, level of cryosectioning 9 layers (0.45 mm) dorsal. **(C)** Metacarpal part of the joint with an ulnar enosseal branch. Note the difference between the diameter values of the same radial main lateral artery depending on measurement’s localization. arrowhead, enosseal branch; eb, enthesial branch; en, enthesis; mcp, metacarpal; mla, main lateral artery; p, phalanx; R, R-branch; U, U-branch.

The arterial diameters were measured in both the metacarpal (Figure 6) and the phalangeal territories. Attention was paid proximally to the R- and U-branches and their primary branches as the MLAs, DA and the arcade of the dorsal triangle, respectively. The R- and U-branches and the MLAs were measured next to their origin. The diameter of both arcades—also in cases when no anastomosis present—was recorded on both the radial and ulnar sides next to the midline of the metacarpal. Finally, the enthesial and bone supplying arteries were also measured. Distally, measurements were undertaken on the palmar plate artery, tenosynovial branch, phalangeal arcade and the supplying branches of the base of the proximal phalanx. The arcade was measured on both the radial and ulnar side of the midline of the phalanx. All the other vessels were measured at their origins. Data are summarized in Supplementary Table 1.

### Ultrasonographic mapping on healthy volunteers

**FIGURE 7:**
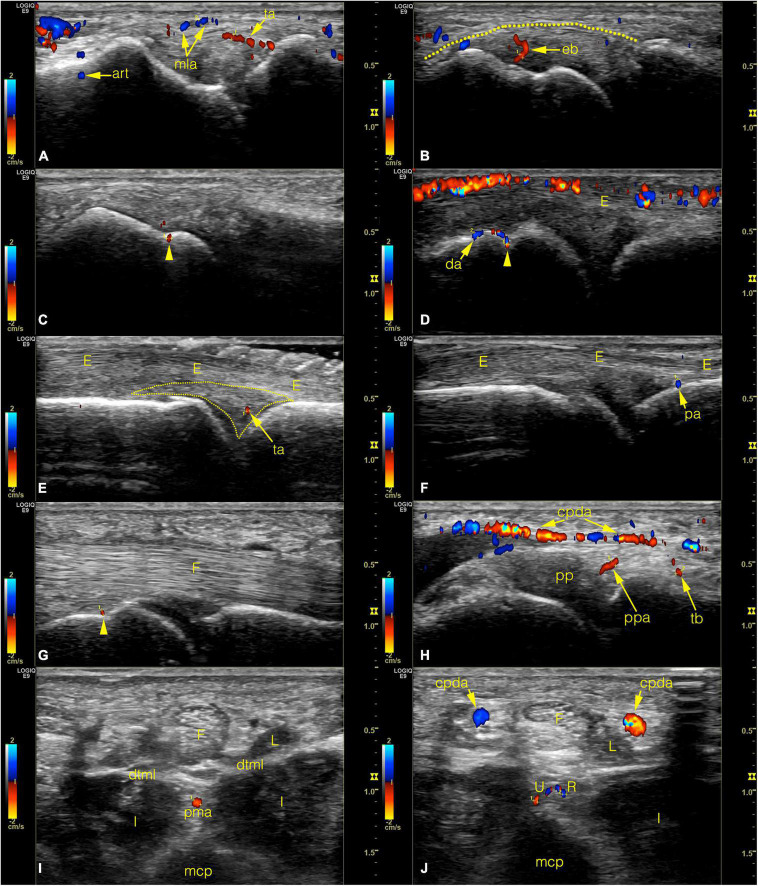
Color Doppler ultrasonographic images of normal joint vessels on healthy volunteers. (Left side of the images refers to the metacarpal half.) **(A–C)** radial side of the 2nd joint on coronal plane. On panel **(B)** the dotted line indicates the outer border of the enthesis. **(D–F)** dorsal side of the joint on sagittal plane images. Note the superficial cutaneous vein along the top of panel **(D)**. On panel **(E)** the dotted line indicates the dorsal triangle. **(G,H)** palmar side on sagittal plane. **(I,J)** axial plane images with the homuncule shaped soft tissue complex in the center with palmar metacarpal artery and it’s bifurcation into R- and U-branches, respectively. arrowhead, entry point of a bone supplying vessel; art, Doppler mirror artifact; cpda, common palmar digital artery; da, dorsal arcade; dtml, deep transverse metacarpal ligament; eb, enthesial branch; E, extensor tendon; F, flexor tendon; I, interosseous muscle; L, lumbrical muscle; mcp, metacarpal; mla, main lateral artery; pa, phalangeal arcade; pma, palmar metacarpal artery; pp, palmar plate; ppa, palmar plate artery; ppda, proper palmar digital artery; R, R-branch; ta, triangular arcade; tb, tenosynovial trunk; U, U-branch.

The MCP joints 2–5 of both hands of two males (ages 31 and 59 years, mean 45 years) and eight females (ages 21–76 years, mean 48.25 years) were scanned as described above (section “Scanning method”). Three joints (1 MCP4 and 2 MCP5) were excluded due to technical problems leaving a total of 77 joints examined using color Doppler mode (Table 1 and Figure 7). The number of recorded images of the left hand ranged between 56–238 (average: 146.6) and 78–266 (average: 139.7) on the right hand, respectively. Supplementary Table 2 summarizes the number of joint specimens with Doppler-signal and their diameters. Due to the lack of proper acoustic window no data were registered on the interdigital surfaces of the joints. The most frequent location with detected Doppler signal was the dorsal depression of the metacarpal head (64.94%) (Figure 7D) and the location of the main lateral arteries (68.42%) (Figure 7A). The distal PMA was identified in 53.25% of the joints, typically embedded in a homunculus-shaped connective tissue mass on axial plane images (Figures 4I, 7I). In all other locations, Doppler signal was captured in less than 50% of cases (Supplementary Table 2).

#### Difference in Doppler signal among metacarpophalangeal joints

**FIGURE 8:**
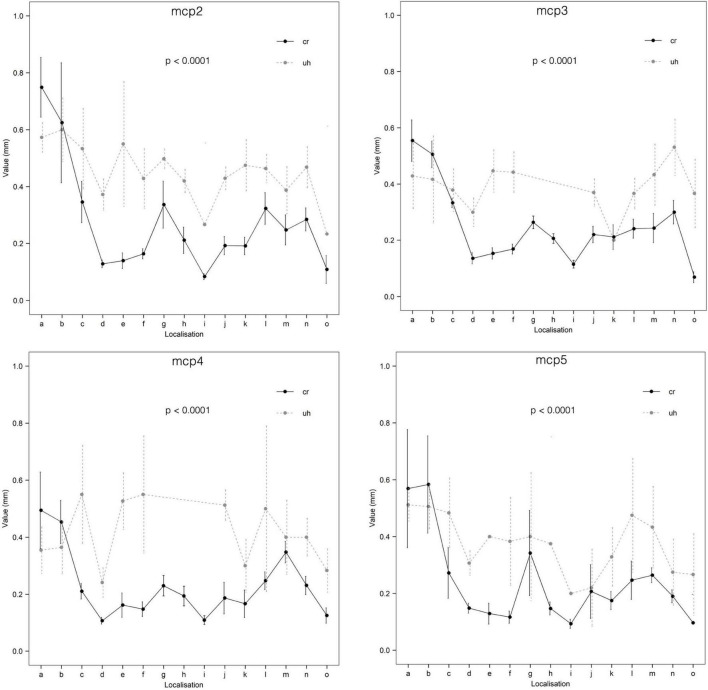
Comparison of ultrasonographic and cryosectioned diameter measurements (CI = 95%). cr, cryosectioning; uh, ultrasonography; a, R-branch; b, U-branch; c, dorsal arcade; d, dorsal enosseal; e, palmar enosseal; f, dorsal triangle arcade; g, main lateral artery; h, enthesial; i, enosseal lateral; j, palmar plate radial; k, palmar plate ulnar; l, tenosynovial branch radial; m, tenosynovial branch ulnar; n, phalanx arcade; o, phalanx dorsal enosseal.

Doppler signal (any) could be identified more frequently in MCP joints 2–3 (MCP2: 136/320, MCP3: 68/260), as compared MCP joints 4–5 (MCP4: 51/247, MCP5: 64/288) (*p* < 0.0001). Comparing the numbers of the intraarticular vessels (enosseal, enthesial and palmar plate) successfully identified with ultrasound, this difference was also observable between MCP joints 2–3 (MCP2: 54/160, MCP3: 29/120) and 4–5 (MCP4: 23/114, MCP5: 24/144) (*p* = 0.009). However, such difference was not present (*p* = 0.1373) between these joints when comparing the great, extraarticular vessels (R-branch, U-branch, main lateral artery). Difference between the vessel diameters measured with ultrasound and on the cryosectioned specimens was significant in all joints getting higher values when measured with ultrasound (*p* < 0.0001) (Supplementary Table 2 and Figure 8).

## Discussion

This study describes the arterial supply of MCP joints and compares anatomic data to *in vivo* Doppler imaging on healthy volunteers. Although various imaging techniques are capable of depicting inflammation within and surrounding the metacarpophalangeal joints and fingers (21, 23), none of them can detect inflammatory mediators, inflammatory cells or the normal synovial lining. However, they can capture and visualize synovial hyperplasia, normal and abnormal vessels to a certain extent (24). One study showed synovial vascularization with Doppler ultrasound corresponding to normal vessels in healthy wrists, first carpometacarpal joints and less frequently in MCP joints (20). Another study raised attention to possible misinterpretation of Doppler-artifacts outside of healthy tendon sheaths on the wrist, 2nd and 3rd fingers, respectively (25). Further studies emphasized that synovial hyperplasia and locally altered vascularization are both important parameters to define and score synovitis (13). On the other hand, the threshold between normal detectable Doppler signal (which represents normal vessels, or normal variants) and pathologic Doppler signal (representing abnormal vessels and flows) remains unknown (20). Padovano et al. described the presence of effusion, synovial hyperplasia or low-grade power Doppler signal in some MCP joints in a large cohort of healthy subjects, emphasizing the need to distinguish between physiologic and pathologic ultrasound findings at the level of the hand joints (19). High-end ultrasonographic equipment is also a validated tool when distinguishing between vascular channels, bony erosions and pseudoerosions in many cases of RA patients and healthy subjects, respectively (26, 27). However, the reliability of ultrasonographic differential diagnostic depends on a lot of factors (e.g., site, size, shape, and scenery as the “four S”) making the decision difficult especially in early RA and young people (27). Considering our results, how to interpret based on the site and size any cortical interruption remains already a question. As it is highlighted in our study, in case of metacarpal heads the vessels enter typically on all four sides the bone, what can be in overlap with erosions site. Finzel et al. described with ultrasonography more false positive results of bony erosions on the palmar aspect of metacarpal heads when comparing them with micro CT images, which was explained with the presence of vascular channels misinterpreted with ultrasound (28). However, we detected anatomically a generally higher number of entering vessels on the dorsal side, which we also confirmed with the much higher number of detected Doppler signals in the same location compared to the palmar side. Despite performance of nowadays ultrasound machines, there is no definite cut-off level for secure differentiation between a lesion and a physiologic vascular channel. As both our anatomical and ultrasonographic measurements confirmed, the size of bone entering vessels remains consequently under 0.7 mm. This fact should be taken into account when examining cortical brakes based on their diameter.

In the past, several anatomical and surgical studies investigated the vascular supply of MCP joints (29, 30). None of these methods provided true *in situ*, high resolution layer-by-layer investigation of the entire joint vasculature. To this date there are no published studies that describe the periarticular, articular arteries (intraenthesial, tenosynovial branches, the dorsal triangle arcade and the phalangeal arcade). There are also no studies that match vascular sonographic findings with the exact anatomy of these articular and periarticular vessels. The cryosectioning method for *in situ* visualization of different human and veterinary tissues has been available for a long time (31–34). We have improved several phases of this method, resulting in a higher resolution (22), which is comparable to histological examination (35). The ultrathin layer technique utilized in our study allowed the precise mapping of very small branches independent of their original three-dimensional course, and enabled computer-aided reconstruction and measurements of them.

The ultrasonographic measurements were carried out on healthy volunteers by the same investigator under standardized circumstances (constant room temperature, warm bath to heat the joints before examination) (36, 37). We chose color Doppler rather than power Doppler because this has been shown to be more sensitive on our ultrasound machine (8). The ultrasonographic settings were based on the guidelines described by Torp-Pedersen et al. (36). In their recent publication, they highlighted that manual settings improved the Doppler sensitivity by an average of 78% and a maximum of 273% over factory settings. Therefore, our machine was calibrated by a professional GE technician with special emphasis on small vessel detection. The timing of ultrasonographic investigations depended on availability of the volunteers, thus the scans were carried out between 7 am and 10 pm. Although Semerano et al. reported higher Doppler signals in MCP circulation of rheumatoid arthritis patients in the morning, this circadian change is likely due to periodic changes in inflammation, because it correlated well with the patient’s symptoms (38). Therefore, it is highly unlikely that the timing of our ultrasonographic investigations had any effect on the variability of our results because our healthy volunteers had no rheumatological complaints on their hands.

Our study revealed that in healthy volunteers, small intraarticular vessels adjacent to the bony cortex or joint space can be detected by ultrasound significantly more frequently in the MCP 2–3 joints as compared to MCP 4–5 joints. This finding is in line with the increased frequency of the involvement of MCP 2–3 in inflammation compared to MCP 4–5 joints seen in RA patients (39).

Our study has also some limitations. Both the comparison of inevitably different joint specimens using post mortem cryosectioning and *in vivo* ultrasonography and the consideration of possible anatomical variations could necessitate a higher number of cadaver specimens and healthy controls, respectively. However, our detailed anatomical mapping on the joint arteries reviled a rather constant pattern of vascularity, and the ultrasonographic examinations were all carried out based on these morphological results. Furthermore, only one ultrasound machine was used by only one examiner. The choice of the applied high-end machine based on the fact, that both the Doppler modality and the calibration data for flow investigation in joints were tested and published in detail previously (8, 36). As the localization of possible vascular signals was clearly determined by the anatomical part of this study, and the investigations were carried out under predefined criteria with no limit on scanning time, no second examiner was invited to the ultrasonographic part. In a future investigation a large pathological group consisting of different inflammatory diseases compared to a higher number of healthy volunteers using different ultrasound machines by more examiners could serve valuable data on (mis)interpretation possibilities of joint blood flow under different clinical conditions.

In summary, we described the entire arterial vasculature of MCP 2–5 joints on anatomical specimens divided it in metacarpal and phalangeal territories, peri- and intraarticular branches. We found that Doppler signal could be detected in only less than 50% of the vessels of healthy volunteers, however the detection probability of the dorsal enosseal branches and the main lateral arteries were much higher. Intraarticular branches were detected with ultrasound imaging significantly more frequently on MCP 2–3 joints. Our findings using ultrasound imaging provide the first reference data for MCP joints Doppler signal appearance and measurements on morphological bases. Our study also provides reference data for future, higher resolution imaging techniques.

## Data availability statement

The original contributions presented in this study are included in the article/Supplementary material, further inquiries can be directed to the corresponding author.

## Ethics statement

Ethical review and approval was not required for the study on human participants in accordance with the local legislation and institutional requirements. Written informed consent for the use of donated human cadaver tissue was not required in accordance with the national legislation and the institutional requirements. All volunteers provided written informed consent to participate in this study.

## Author contributions

GB contributed to the study design. GB, KC, OP, and SB prepared the anatomical specimens. GB, KC, OP, JG, SH, and GN collected the data. GB, VS, PM, PB, and KC wrote the first draft of the manuscript. GB and LB made all the statistical examinations and designed the figures and tables. All authors revised the manuscript critically and approved the final version of the manuscript.

## Footnotes

1
www.adobe.com

2
www.fei.com
