# Supplementary material for: Vascular supply of the metacarpophalangeal joint
Source: Front Med (Lausanne). 2022 Oct 20;9:1015895. doi: 10.3389/fmed.2022.1015895 (PMC9630748; doi:10.3389/fmed.2022.1015895)
Supplement: Supplementary file 1 [file Table_1.docx]

Supplementary Material

| **Σ_ν_=12** | **MCP2** | **MCP3** | **MCP4** | **MCP5** |
| --- | --- | --- | --- | --- |
|  | avg. [min - max.] | avg. [min - max.] | avg. [min - max.] | avg. [min - max.] |
| **Metacarpal territory** |  |  |  |  |
| **R-branch** | 0.749 [0.577-0.860] | 0.554 [0.433-0.647] | 0.494 [0.353-0.717] | 0.569 [0.357-0.927] |
| **U-branch** | 0.624 [0.407-0.987] | 0.506 [0.450-0.573] | 0.453 [0.323-0.527] | 0.583 [0.297-0.787] |
| **Main lateral - radial** | 0.334 [0.267-0.407] | 0.277 [0.223-0.333] | 0.240 [0.203-0.270] | 0.546 [0.297-0.963] |
| **Main lateral - ulnar** | 0.298 [0.273-0.327] | 0.245 [0.237-0.253] | 0.220 [0.137-0.343] | 0.139 [0.110-0.170] |
| **Dorsal arcade - radial** | 0.440 [0.377-0.503] | 0.333 [0.317-0.357] | 0.243 [0.213-0.287] | 0.398 [0.227-0.597] |
| **Dorsal arcade -ulnar** | 0.205[0.160-0.250] | 0.198 [0.120-0.247] | 0.178 [0.123-0.237] | 0.147 [0.090-0.257] |
| **Dorsal triangle radial** | 0.141 [0.120-0.167] | 0.161 [0.147-0.180] | 0.154 [0.103-0.217] | n.i. |
| **Dorsal triangle ulnar** | 0.186 [0.167-0.203] | 0.176 [0.157-0.207] | 0.141 [0.083-0.180] | 0.117 [0.090-0.143] |
| **Enthesial vessels** |  |  |  |  |
| **radial** | 0.211 [0.160-0.280] | 0.208 [0.187-0.233] | 0.158 [0.133-0.193] | 0.174 [0.140-0.230] |
| **ulnar** | 0.211 [0.150-0.243] | 0.204 [0.170-0.257] | 0.230 [0.137-0.303] | 0.120 [0.080-0.153] |
| **Enosseal vessels** |  |  |  |  |
| **radial dorsal** | 0.134 [0.100-0.160] | 0.143 [0.113-0.167] | 0.101 [0.080-0.127] | 0.148 [0.120-0.170] |
| **ulnar dorsal** | 0.118 [0.110-0.127] | 0.123 [0.087-0.160] | 0.113 [0.097-0.123] | 0.149 [0.113-0.203] |
| **radial palmar** | 0.153 [0.107-0.210] | 0.152 [0.143-0.160] | n.i. | 0.120 [0.067-0.173] |
| **ulnar palmar** | 0.118 [0.080-0.157] | 0.153 [0.087-0.187] | 0.170 [0.113-0.220] | 0.138 [0.087-0.190] |
| **radial** | 0.083 [0.067-0.093] | 0.116 [0.077-0.140] | 0.097 [0.077-0.130] | 0.106 [0.073-0.133] |
| **ulnar** | 0.108 [0.073-0.140] | 0.113 [0.087-0.130] | 0.122 [0.097-0.153] | 0.081 [0.077-0.083] |
| **Phalangeal territory** |  |  |  |  |
| **Palmar plate – radial** | 0.195 [0.150-0.240] | 0.209 [0.187-0.247] | 0.205 [0.127-0.280] | 0.166 [0.127-0.287] |
| **Palmar plate – ulnar** | 0.193 [0.160-0.227] | 0.209 [0.157-0.277] | 0.184 [0.103-0.240] | 0.167 [0.150-0.200] |
| **Tenosynovial branch - radial** | 0.328 [0.257-0.400] | 0.272 [0.197-0.290] | 0.251 [0.213-0.267] | 0.266 [0.187-0.360] |
| **Tenosynovial branch - ulnar** | 0.268 [0.200-0.337] | 0.262 [0.190-0.327] | 0.313 [0.290-0.387] | 0.275 [0.227-0.287] |
| **Phalanx arcade - radial** | 0.352 [0.273-0.430] | 0.362 [0.310-0.423] | 0.313 [0.190-0.387] | 0.253 [0.177-0.270] |
| **Phalanx arcade – ulnar** | 0.220 [0.177-0.263] | 0.236 [0.210-0.277] | 0.240 [0.150-0.333] | 0.192 [0.140-0.197] |
| **Enosseal vessels** |  |  |  |  |
| **radial dorsal** | 0.150 [0.067-0.220] | n.i. | 0.126 [0.087-0.157] | n.i. |
| **ulnar dorsal** | n.i. | n.i. | n.i. | n.i. |
| **radial palmar** | 0.186 [0.180-0.190] | 0.197 [0.170-0.217] | 0.139 [0.103-0.183] | 0.139 [0.117-0.157] |
| **ulnar palmar** | 0.161 [0.147-0.187] | 0.174 [0.127-0.223] | 0.106 [0.077-0.140] | 0.151 [0.130-0.190] |

**Supplementary table 1.** **Arterial diameter measurements on cryosectioned specimens in millimeters.** Abbreviations: n.i.: not identified
