# Supplementary material for: Vascular supply of the metacarpophalangeal joint
Source: Front Med (Lausanne). 2022 Oct 20;9:1015895. doi: 10.3389/fmed.2022.1015895 (PMC9630748; doi:10.3389/fmed.2022.1015895)
Supplement: Supplementary file 2 [file Table_2.docx]

Supplementary Material

| **Σ_ν_=77** | **MCP2 (n=20)** | | **MCP3 (n=20)** | | **MCP4 (n=19)** | | **MCP5 (n=18)** | |  |
| --- | --- | --- | --- | --- | --- | --- | --- | --- | --- |
|  | D_n_ | avg. [min - max.] | D_n_ | avg. [min - max.] | D_n_ | avg. [min - max.] | D_n_ | avg. [min - max.] | D_n∑_ (%) |
| **Metacarpal territory** |  |  |  |  |  |  |  |  |  |
| **R-branch** | 16 | 0.059 [0.040-0.080] | 4 | 0.045 [0.035-0.060] | 6 | 0.036 [0.020-0.050] | 9 | 0.052 [0.043-0.060] | 35 (45.45) |
| **U-branch** | 9 | 0.072 [0.043-0.095] | 4 | 0.041 [0.030-0.060] | 7 | 0.037 [0.027-0.055] | 8 | 0.050 [0.040-0.067] | 28 (36.36) |
| **Dorsal arcade** | 6 | 0.051 [0.040-0.065] | 8 | 0.041 [0.030-0.060] | 4 | 0.059 [0.047-0.080] | 2 | 0.048 [0.047-0.050] | 20 (25.97) |
| **Dorsal enosseal** | 15 | 0.037 [0.020-0.060] | 12 | 0.049 [0.017-0.050] | 9 | 0.023 [0.010-0.040] | 14 | 0.032 [0.017-0.045] | 50 (64.94) |
| **Palmar enosseal - radial** | 2 | 0.022 [0.020-0.023] | 6 | 0.042 [0.037-0.060] | 4 | 0.056 [0.043-0.073] | 0 | 0.000 | 12 (15.58) |
| **Palmar enosseal - ulnar** | 7 | 0.037 [0.020-0.060] | 0 | 0.000 | 1 | 0.040 [0.040-0.040] | 1 | 0.040 [0.040-0.040] | 9 (11.69) |
| **Dorsal triangle arcade** | 7 | 0.040 [0.027-0.067] | 8 | 0.033 [0.030-0.050] | 5 | 0.055 [0.040-0.070] | 4 | 0.043 [0.030-0.060] | 24 (31.17) |
| **Main lateral artery - ^1^radial/^2^ulnar** | ^1^19 | 0.050 [0.040-0.073] | no acoustic window | | no acoustic window | | ^2^7 | 0.044 [0.040-0.055] | 26 (68.42) |
| **Enthesial - ^1^radial/^2^ulnar** | ^1^13 | 0.041 [0.030-0.053] |  |  |  |  | ^2^2 | 0.038 [0.020-0.055] | 15 (39.47) |
| **Enosseal - ^1^radial/^2^ulnar** | ^1^2 | 0.030 [0.020-0.040] |  |  |  |  | ^2^0 | 0.000 | 2 (5.26) |
|  |  |  |  |  |  |  |  |  |  |
| **Phalangeal territory** |  |  |  |  |  |  |  |  |  |
| **Palmar plate - radial** | 7 | 0.042 [0.030-0.050] | 4 | 0.038 [0.033-0.045] | 3 | 0.051 [0.045-0.057] | 2 | 0.021 [0.015-0.027] | 16 (20.78) |
| **Palmar plate - ulnar** | 5 | 0.047 [0.040-0.075] | 1 | 0.020 [0.020-0.020] | 2 | 0.028 [0.020-0.040] | 4 | 0.033 [0.030-0.050] | 12 (15.58) |
| **Tenosynovial branch - radial** | 11 | 0.046 [0.033-0.057] | 3 | 0.038 [0.033-0.040] | 1 | 0.043 [0.030-0.057] | 2 | 0.042 [0.030-0.053] | 17 (22.08) |
| **Tenosynovial branch - ulnar** | 4 | 0.038 [0.030-0.040] | 4 | 0.044 [0.033-0.060] | 2 | 0.037 [0.030-0.043] | 3 | 0.039 [0.020-0.050] | 13 (16.88) |
| **Phalanx arcade** | 10 | 0.047 [0.020-0.070] | 8 | 0.055 [0.037-0.065] | 3 | 0.039 [0.035-0.043] | 5 | 0.033 [0.015-0.050] | 26 (33.77) |
| **Phalanx dorsal enosseal** | 3 | 0.023 [0.010-0.040] | 6 | 0.032 [0.010-0.047] | 4 | 0.028 [0.020-0.040] | 1 | 0.027 [0.027-0.027] | 14 (18.18) |

**Supplementary table 2.** **Color Doppler signals and measurements on healthy volunteers in centimeters.** Abbreviations: D_n_: number of joints, with color Doppler signal; D_n∑_: total number of joints within category where color Doppler signal was detected; MCP: metacarpophalangeal joint
